# Predicting disease-free survival following curative-intent resection of right-sided colon cancer using a pre- and post-operative nomogram: a prospective observational cohort study

**DOI:** 10.1097/JS9.0000000000002300

**Published:** 2025-02-24

**Authors:** James Lucocq, Tom Trinder, Kate Homyer, Hassan Baig, Pradeep Patil, Girivasan Muthukumarasamy

**Affiliations:** a Department of Colorectal Surgery, Ninewells Hospital, Dundee, United Kingdom; b Department of General Surgery, Ayr Hospital, Ayr, United Kingdom; c Department of General Surgery, Royal Infirmary of Edinburgh, Edinburgh, United Kingdom

**Keywords:** colon cancer, prediction, recurrence, right hemicolectomy, survival

## Abstract

**Introduction:**

Disease prognostication can be achieved through the derivation of biologically and clinically integrated prediction models. The present study reports 1-, 3-, and 5-year disease-free survival (DFS) in patients undergoing right hemicolectomy for curative intent and both derives and validates a pre- and post-operative prediction tool for DFS for prognostication and risk stratification purposes.

**Method:**

Consecutive patients undergoing right-sided curative-intent resection for colorectal cancer (2010–2020) in a tertiary care unit were followed-up prospectively for recurrence and survival outcomes. Survival analyses were used to derive pre- and post-operative models predicting 1-, 3-, and 5-year DFS. Calibration was reported and internal validation was performed using bootstrapping.

**Results:**

A total of 822 patients underwent resection and 528 had ≥5-year follow-up. The 1-, 3-, and 5-year DFS rates were 85.6%, 72.5% and 57.6%, respectively. Variables associated with death/recurrence included: increasing age (HR > 1.95, *P* = 0.037), male gender (HR 1.62, *P* < 0.001), ASA ≥3 (HR 1.79, *P* < 0.001), low albumin (HR 1.54, *P* < 0.001), T4 stage (HR 2.35, *P* = 0.023), R1 status (HR 1.63, *P* = 0.024), ≥4 positive lymph nodes (HR > 1.74, *P* < 0.001) and Clavien-Dindo ≥3 (HR 2.83, *P* < 0.001). The pre- and post-operative models contained 9 and 13 demographic, clinical, biochemical, operative and pathological variables, respectively (C-index 0.75 and 0.79, respectively). Excluding demographic, clinical and operative variables significantly reduced the C-index of the pre- (0.62) and post-operative models (0.70).

**Conclusion:**

The presented prediction tools for DFS will help clinicians stratify risk, offer appropriate adjuvant treatment and predict long-term DFS following curative-intent right-sided colon cancer resection.

## Introduction

Colorectal cancer is the second most common cause of cancer death in the United Kingdom and resection remains the mainstay of treatment^[1,2]^. Disease-free survival (DFS) is increasingly being recognized as a valuable outcome metric in colorectal cancer and is commonly being reported in colorectal clinical trials. Five-year DFS rates approach 80% for elective right hemicolectomy^[3-9]^.

**Highlights**

- Demographic, clinical, biochemical, operative and pathological factors influence the likelihood of disease-free survival.
- The pre-and post-operative prediction tools estimate 1-, 3-, and 5-year disease-free survival with good accuracy following curative-intent right hemicolectomy.
- The models have a role in risk stratification, offering appropriate adjuvant treatment and predicting long-term survival.

Colorectal adenocarcinomas are currently staged and managed based on the TNM (Tumor, Nodes, Metastasis) classification of solid tumors in adults, developed by the Union for International Cancer Control (UICC) and used by the American Joint Committee on Cancer (AJCC). TNM staging is used to plan management and predict outcomes such as recurrence by grouping patients based on their stage and applying outcomes from clinical trials specific to the type and site of cancer. Predicting outcomes based on median values derived from clinical trials is not patient centric or individualized; patients with the same TNM stage cancer can have varying survival ^[10,11]^. The TNM staging relies only on the tumor stage and does not take other patient characteristics into consideration such as asymptomatic patients (screen-detected), age, comorbidities and both operative and perioperative outcomes, which may have a bearing on long-term DFS. In other words, individualized outcome prediction is lacking for cancer patients. This information, if available will better inform both clinicians and patients while enabling shared decision making regarding appropriate modalities of care. There is currently no known method of predicting cancer recurrence or survival specific to right colon cancers^[12-16]^.

The aim of this study is to develop preoperative and postoperative prediction models for long term (5 years) survival for patients with right sided colon cancer. Patient-specific preoperative prediction models will help shared decision-making regarding treatment modalities. Postoperative model adding perioperative complications and tumor pathological characteristics will estimate risk of recurrence which can guide follow up intensity and need for adjuvant treatments. The models are intended to be simple, user friendly and applicable to all general hospitals throughout the world whilst demonstrating strong statistical ability to predict outcomes in individual patients.

## Methods

### Study design

We undertook an observational, cohort study of patients undergoing right-sided hemicolectomies for colorectal cancer from January 2010 to December 2020 in a tertiary care unit, reported according to the Strengthening the Reporting of Observational Studies in Epidemiology (STROBE)^[17]^. Data usage approval was granted by our institutional review board and the study was conducted in accordance with the Declaration of Helsinki. The manuscript has been reported in line with the STROCSS criteria^[18]^.

All patients aged over 18 years undergoing right hemicolectomy for colorectal cancer were identified from institutional electronic records. The following patients were excluded: procedures not performed for curative intent (*n* = 92), patients who died in the immediate post-operative period (<30 days, *n* = 24), those with metastasis (*n* = 42), those without adenocarcinoma on pathology (*n* = 29) and distal transverse tumors (*n* = 70).

### Data variables

Background patient characteristics were documented such as demographics, comorbidities, American Society of Anesthesiologists (ASA) score, metabolic equivalent (MET) score, smoking history, body mass index (BMI), colon cancer symptoms, presentation (e.g. emergency, elective, or screen-detected), and referral pathway. The type of right-sided colonic resection (e.g. total mesentery excision, extended) was performed at the discretion of the surgeon and based on the clinical suspicion and local invasion of the tumor. The surgical approach (laparoscopic versus open), the need for conversion, the urgency of the procedure and the need for a stoma were documented.

Histopathological data were extracted from postoperative pathology reports and malignant tumors were included. The histological type of cancer (e.g. mucinous adenocarcinoma) was noted, as well as the differentiation, lymphovascular invasion, T and N stage, number of lymph nodes in the specimen and the number of positive lymph nodes. The resection “R1” status was determined on a cut-off distance from the tumor to the resection margin of <1 mm. Contrast-enhanced computed tomography (CT) of the chest, abdomen and pelvis were used to determine the stage of each patient. The use of adjuvant chemotherapy was reported.

### Outcomes

The diagnosis of recurrence was made by radiological or histological diagnosis and MDT review. The site of recurrence was classified as either loco-regional or systemic. The length of follow-up for each patient was recorded and the date or recurrence or death, if applicable, were recorded. The time from the date of surgery to the date of the first event (recurrence or death), was recorded. Data was censored for patients without recurrence and still alive at the end of follow-up. The rate of recurrence and DFS were reported using Kaplan–Meier (KM) curves.

KM curves were used to determine variables associated with DFS using the log-rank test accounting for the length of follow-up. In the log-rank analysis, patient age was categorized by decade, BMI was divided by 25 kg/m^2^, ASA was dichotomized by a cut-off of 1–2 versus ≥3 and METs score was divided by 1–4 versus ≥5. Low Albumin was defined as <35 g/dL and a low Hb was <120 g/dL for females and <130 g/dL for males. The number of lymph nodes in the specimen were reported and the number of positive lymph nodes were grouped as follows: 0; 1–3; 4–5; 6–10; >10. For all statistical tests, patients with missing data were excluded from analyses. Cox-Proportional Hazards Models (CPHM) were used as a multivariate approach to determine variables associated with DFS. Factors considered for inclusion in the CPHM were determined from previous studies and by a *P* < 0.1 demonstrated from the logrank analysis.

### Long-term (5 year) disease-free survival

To demonstrate the association of variables with long-term DFS (5-year) a subgroup analysis was performed. Of those with 5-year follow-up, variables associated with 5-year DFS were determined. Baseline categorical data were presented and analyzed using the independent samples *X*^2^ test. Continuous data were assessed for normality and managed accordingly using a one-way ANOVA or Kruskal–Wallis test as appropriate.

### Developing predictive model for 1-, 3-, and 5-year disease-free survival

We adhered to the AJCC criteria for developing risk models during the development of our risk model^[8]^. Variables were included in the initial CPHM model based on significant variables (*P* < 0.1) in the log-rank analysis. Two separate models were created: a model derived from all variables accessible pre-operatively and a further post-operative model derived from variables available once the final specimen pathology was available.

Resulting hazard ratios (HR’s) and *P*-values for the CPHM were reported. Akaike’s information criterion (AIC) was minimized to select the final prediction model using a backward elimination process. Variance inflation factor (VIF) was used to assess for multicollinearity between variables and a value greater than 10 indicated significant collinearity. The concordance index (C-index) was used to determine discriminative ability for the training and test cohort, using the “rms” package in R studio 2022.02.01. The model was trained using 1000 bootstrapped samples and then correction for model optimism was performed by internal validation on out-of-sample test data. Calibration was assessed using calibration plots on bootstrap resampling data to simulate an out-of-sample validation. The models were displayed as nomograms.

Sensitivity analysis was assessed by plotting receiver operator characteristic curves and assessing the area under the curve. To demonstrate the impact of adding demographic, clinical and operative variables into the predictive model in addition to the pathological variables, the C-index was reported for models with and without the addition of demographic, clinical and operative variables.

## Results

**Figure 1.**
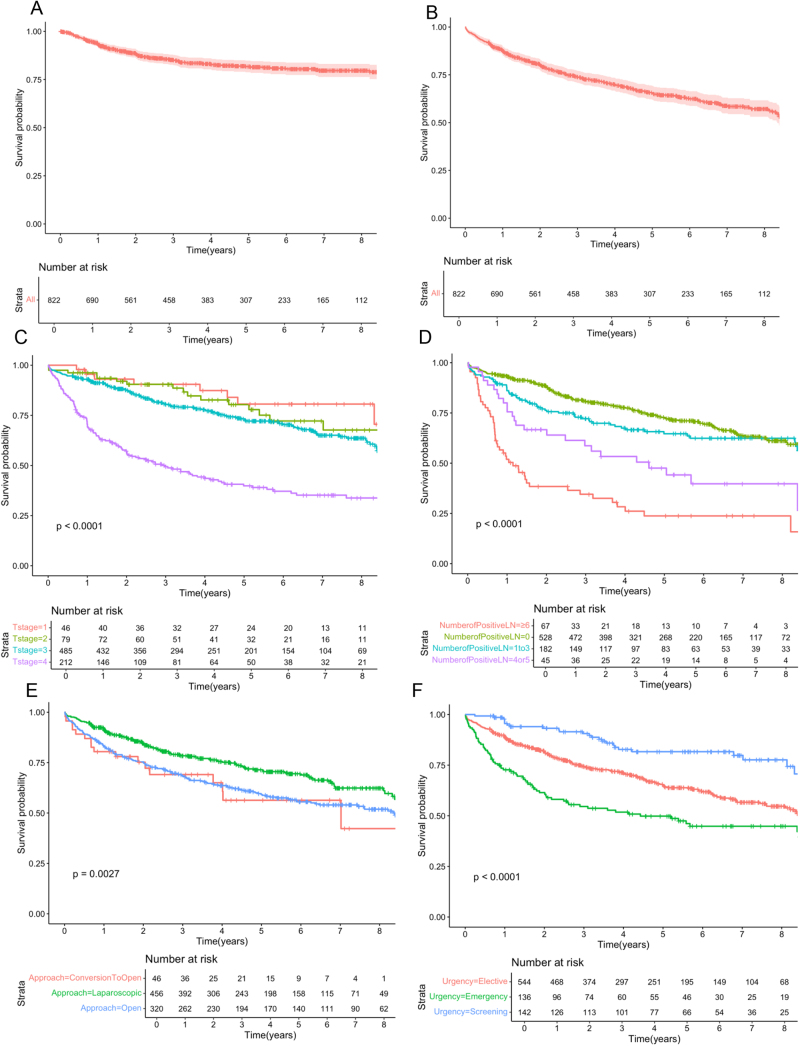
(A) Recurrence and (B) DFS during follow-up. (C) DFS based on number of positive lymph nodes. (D) DFS by T stage. (E) DFS by urgency of procedure. (F) DFS by operative approach.

A total of 822 patients (median age, 73; IQR, 58–82) with malignant right-sided colon cancer undergoing resection with curative intent were included (Table 1). Figure 1 demonstrates the risk of recurrence (Fig. 1A) and DFS (Fig. 1B) over time after a median follow-up of 5 years and 7 months. The 1-, 3-, and 5-year recurrence rates were 7.2%, 14.5%, and 17.6%, respectively (Table 2). The 1-, 3-, and 5-year DFS rates were 85.6%, 72.5% and 57.6%, respectively.

**Table 1:** Clinicopathological data and association with recurrence/death (inverse of DFS) with survival analyses (KM and CPHM).

Variable		All patients,	Logrank analysis	CPHM
		*N* = 822, %		HR, 95%CI
				(*P*-value)
Median Age (years)^a^		73	<0.001	1.95, 1.03–3.68 (0.037)
Male		387 (47.1)	0.003	1.62, 1.26–2.08 (<0.001)
Median BMI^b^		27.2	0.056	–
Median ASA		2	<0.001	1.79, 1.34–2.38 (<0.001)
Smoker		87 (10.5)	0.132	1.41, 0.97–2.04 (0.073)
Comorbidities	IHD	441 (53.6)	<0.001	0.92, 0.70–1.21 (0.598)
	DM	150 (18.2)	<0.001	0.96, 0.70–1.30 (0.715)
	CKD	62 (7.5)	<0.001	0.75, 0.50–1.11 (0.367)
	COPD	96 (11.7)	0.312	–
	Stroke/CNS dysfunction	86 (10.4)	0.028	–
Median METS score		7	<0.001	*
Symptoms	Abdominal pain	124 (15.1)	0.044	0.89, 0.59–1.35 (0.383)
	Anaemia	358 (43.6)	0.485	–
	Obstruction	35 (4.3)	<0.001	1.17, 0.63–2.16 (0.740)
	Change in bowel habit	71 (8.6)	0.908	–
	Other	251 (30.5)	–	Reference
Median pre-operative hemoglobin (g/dL)		12.1	<0.001	1.18, 0.91–1.53 (0.102)
Median pre-operative albumin (g/dL)		35	<0.001	1.54, 1.17–2.02 (0.008)
Pre-operative diagnosis	Caecum tumor	265 (32.2)	<0.001	Reference
	Ascending tumor	289 (35.2)		1.02, 0.76–1.36 (0.826)
	Transverse tumor	185 (22.5)		0.79, 0.58–1.08 (0.069)
Urgency	Elective	544 (66.2)	<0.001	Reference
	Emergency	136 (16.5)		1.07, 0.68–1.69 (0.757)
	Screening	142 (17.3)		0.61, 0.39–0.96 (0.109)
Operative approach	Laparoscopic	410 (49.9)	0.001	Reference
	Laparoscopic converted to open	46 (5.6)		1.18, 0.69–2.03 (0.362)
	Open	366 (44.5)		1.07, 0.81–1.40 (0.941)
Extended right hemicolectomy		181 (22.0)	0.20	–
Median operation length		2.92	0.564	–
Stoma		29 (3.5)	0.001	1.67 0.93–3.02 (0.345)
Clavien-Dindo 2		200 (24.3)	<0.001	1.06, 0.79–1.42 (0.489)
Clavien-Dindo ≥3		66 (8.0)		2.83, 1.93–4.14 (<0.001)
Differentiation	Well	33 (4.0)	0.014	Reference
	Moderate	609 (74.1)		1.39, 0.72–2.70 (0.121)
	Poor	180 (21.9)		1.63, 0.81–3.27 (0.055)
Lymphovascular invasion		325 (39.5)	<0.001*	1.37, 1.04–1.80 (0.122)
T stage	1	46 (5.6)	<0.001	Reference
	2	79 (9.6)		0.98, 0.41–2.34 (0.991)
	3	485 (59.0)		1.08, 0.68–1.71 (0.795)
	4	212 (25.8)		2.35, 1.42–3.89 (0.023)
Lymph nodes in sample	Less than 12	43 (5.2)	0.048	Reference
	12–19	330 (40.1)		0.74, 0.44–1.25 (0.295)
	20–29	315 (38.3)		0.54, 0.32–0.92 (0.032)
	30 and over	134 (16.3)		0.44, 0.23–0.81 (0.012)
Positive lymph nodes	1–3	182 (22.1)	<0.001	1.08, 0.78–1.50 (0.486)
	4–5	45 (5.5)		1.74, 1.10–2.78 (0.012)
	6–10	46 (5.6)		3.65, 2.33–5.73 (<0.001)
	>10	21 (2.5)		3.86, 2.12–7.02 (<0.001)
R1 status		39 (4.7)	0.004	1.63, 1.05–2.53 (0.024)
Adjuvant chemotherapy		226 (27.5)	0.097	0.76, 0.53–1.09 (0.141)

^a^ Age 50–59, age 60–69, age 70–79 and age ≥80 were all positively associated with recurrence/death.

^b^ Not included in multivariate analysis as not available in all patients.

**Table 2:** Recurrence and DFS outcome data for entire cohort.

Outcome measure		Number
Recurrence	Overall	17.0% (140/822)
	1 year	7.2% (59/822)
	3 year	14.5% (90/622)
	5 year	17.5% (94/536)
Locoregional recurrence		3.0% (25/822)
Systemic recurrence		15.5% (127/822)
DFS	Overall	63.4% (521/822)
	1 year	85.6% (704/822)
	3 year	72.5% (451/622)
	5 year	57.6% (304/528)

As demonstrated in the KM analysis, a multitude of variables were associated with recurrence and/or death (Table 1). Figure 1 displays KM analyses for DFS based on T stage (Fig. 1C), number of positive lymph nodes (Fig. 1D), operative approach (Fig. 1E), and urgency of procedure (Fig. 1F).

In CPHM, a number of variables were positively associated with recurrence/death: increasing age (HR > 1.95, *P* = 0.037), male gender (HR 1.62, *P* < 0.001), ASA ≥3 (HR 1.79, *P* < 0.001), low albumin (HR 1.54, *P* < 0.001), T4 stage (HR 2.35, *P* = 0.023), R1 status (HR 1.63, *P* = 0.024), 4–5 positive lymph nodes (HR 1.74, *P* < 0.001), 6–10 positive lymph nodes (HR 3.65, *P* < 0.001) and Clavien-Dindo ≥3 (HR 2.83, *P* < 0.001). The following variables were negatively associated with recurrence and/or death: 20–29 lymph nodes in the specimen (HR 0.54, *P* = 0.032) and ≥30 lymph nodes in the specimen (HR 0.44, *P* = 0.012). No variables suffered from significant multicollinearity (VIF > 10).

### Long-term (5 year) disease-free survival

The subgroup of patients (*n* = 528) who were followed up for at least 5-years were split into two groups: those with (*n* = 304) and without (*n* = 224) 5-year DFS. The clinicopathological characteristics were compared between the two groups and are reported in Supplementary Digital Content (Table 1, http://links.lww.com/JS9/D966). Patients who survived 5 years without recurrence were younger, less likely to be male, had lower rates of diabetes mellitus (DM), chronic kidney disease (CKD) stage ≥3, ischaemic heart disease (IHD), higher albumin levels and were more likely to be identified during screening but less likely to have undergone an emergency hemicolectomy (Supplementary Digital Content, Table 1, http://links.lww.com/JS9/D966). Further significant differences between the two groups include the 5-year DFS group having lower numbers of positive lymph nodes, greater number of resected lymph nodes and lower overall T and N stages.

### Developing a pre-operative and post-operative model for 1- and 5-year disease-free survival

**Figure 2.**
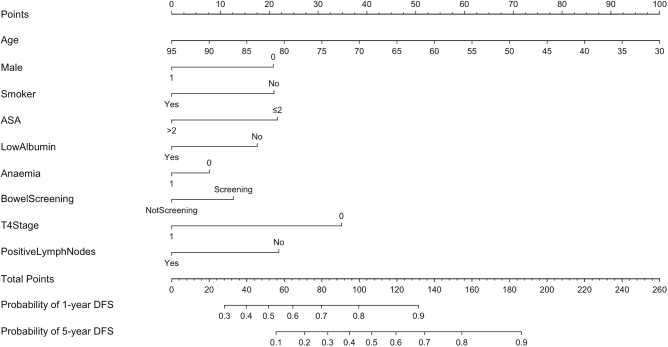
Preoperative nomogram to predict likelihood of 1-, 3-, and 5-year DFS.

Based on the AIC, 9 pre-operative variables were selected for the final pre-operative prediction model and were used to predict 1-, 3-, and 5-year DFS (Fig. 2). Using the model for an individual patient, the probability of DFS can be calculated by summing together the points derived from each variable. The C-index for the pre-operative model was 0.75 and once adjusted for internal validation, the C-index was 0.74 with a slope of 0.84. The calibration plots for 1-, 3-, and 5-year DFS outcomes are reported (Supplementary Digital Content, Figure 1, http://links.lww.com/JS9/D899). Once excluding demographic, clinical and operative variables and only including the pre-operative pathological variables, the C-index for the internal validation was 0.62.

**Figure 3.**
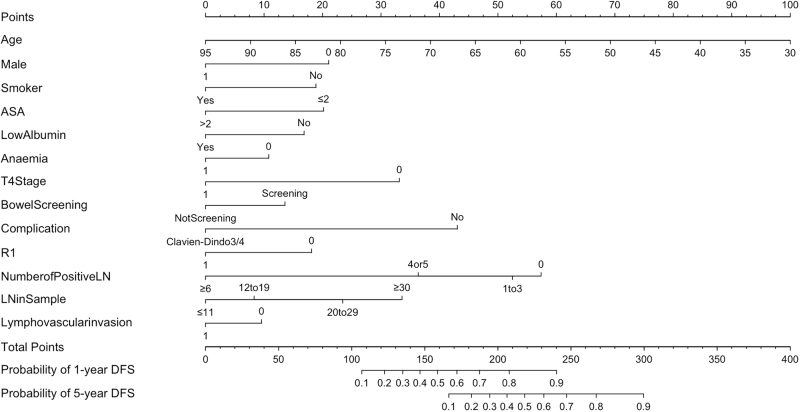
Postoperative nomogram to predict likelihood of 1-, 3-, and 5-year DFS.

Based on the AIC, 13 variables were selected for the final optimal post-operative prediction model and were used to predict 1-, 3-, and 5-year DFS (Fig. 3). The C-index for the model is 0.79 and once adjusted for internal validation, the C-index was 0.77. The slope of the model was 0.86. The calibration plots for 1-, 3-, and 5-year DFS outcomes are reported for the post-operative model (Supplementary Digital Content, Figure 2, http://links.lww.com/JS9/D900). Once excluding demographic, clinical and operative variables and only including the post-operative pathological variables, the C-index for the internal validation model was 0.70.

## Discussion

The present study reports oncological and survival outcomes for patients undergoing a right-sided colonic resection for malignancy with curative intent and identifies variables that are associated with long-term (5-year) DFS. We derive and validate pre- and post-operative prediction tools for 1-, 3-, and 5-year DFS that accounts for demographic, clinical, biochemical, operative and pathological factors. Incorporating all variables boosts the predictive ability for DFS compared to pathological factors alone.

The present prediction tool determines the factors that independently predict outcome, many of which are well known to be linked to recurrence or survival. For example, the number of lymph nodes resected has been demonstrated as a prognostic factor in right hemicolectomy^[19,20]^, as are T stage, N stage and the presence of lymphovascular invasion^[19]^. Less well understood is how such factors culminate in an overall risk of recurrence or death. By aggregating these factors, we gain an appreciation of the numerous aspects that influence prognosis and a more accurate estimation of the long-term outcome. Whilst the post-operative model has the highest utility once the formal specimen pathology is reported, both the pre-operative and post-operative models demonstrate high predictive ability. The post-operative model has a role in counseling patients on prognosis and long-term follow-up. Utilizing the pre-operative model has a role in patient counseling on the peri-operative risk and can help clinicians decide between surgical candidates, particularly in high-risk patients^[21]^.

To date, the derivation of predictive models for survival outcomes following the resection of right-sided colon cancer for curative intent has not been conducted. Increasing evidence supports the disparate patient characteristics and outcomes of right and left colectomy, more specifically, the worse prognosis, higher rate of post-operative complications and longer length of hospital stay following right hemicolectomy as well as the differing response to adjuvant chemotherapy^[12-16]^. There are significant genetic differences between right and left sided cancers, such as an association of microsatellite instability (MSI) with right sided cancers, compared to the chromosomal instability pathway mutations on the left^[22,23]^. The above is evidence that predictive models for right and left-sided cancers should be derived and implemented independently.

Current predictive models in patients undergoing colectomy have aimed to predict recurrence or cancer-specific survival following colectomy^[24,25]^. Although previous models incorporate pathological variables (e.g. T stage, number of positive lymph nodes, adjuvant chemotherapy) similar to the present study, there are fundamental differences.^[3-7,24]^. The MSKCC clinical calculator aims to predict recurrence but censors for patients who have died without recurrence^[24]^. As a model orientated towards pathological factors that may be associated with recurrence it does not account for survival or acknowledge clinical or operative factors. Furthermore, a pre-operative prediction of outcome is not provided. Zheng *et al* have created a prediction tool to predict cancer-specific survival^[25]^. This model does not predict recurrence or mortality that is not directly attributed to cancer. After accounting for a multitude of aspects such as demographic factors, significant comorbidities, operative factors, biochemical factors and pathological factors the present study uses DFS as an overall outcome metric that incorporates both recurrence and survival whilst providing pre- and post-operative estimates.

With the emergence of molecular phenotypes and biomarkers future studies should consider how these could be incorporated into models to help improve prognostication further, particularly in conjunction with targeted therapies^[26]^. The presence of MSI and both KRAS and BRAF genes help inform prognosis but also response to adjuvant treatment. For example, MSI Stage II CRC patients do not appear to benefit from 5-FU chemotherapy, but may respond to checkpoint inhibitors^[27-29]^. Circulating tumor DNA may also offer prognostic information pre- and post-operatively and may be monitored in response to treatment^[30,31]^. Of course, there are a number of barriers to overcome before universal use by all healthcare providers can be achieved, such as lack of technical standardization between laboratories, low numbers of validating studies and inadequately-sized patient cohorts.

There were a number of variables which were not explored in the present study. Pre-operative CEA has been shown to be predictive of recurrence in univariate analysis and could hold predictive value. Post-operative CEA reportedly offers even more value in predicting recurrence and could help improve the prediction of DFS during the post-operative period^[32]^. Other factors which were not considered include perineural invasion and tumor infiltrating lymphocytes. Although perineural invasion was not considered, it is unlikely to have influenced the results significantly due to its high correlation with lymphovascular invasion. Whilst molecular phenotypes and biomarkers were not used in the present model, the included variables should be available to the vast majority of pathology and surgical departments which improves the generalizability of the models^[33,34]^.

There were a number of considerations when constructing the present models^[35]^. Including too many variables can result in overfitting of the model to our data cohort. Secondly, with numerous variables particularly in the final model, it was important that we assessed for multicollinearity to ensure appropriate model specification. Multicollinearity could falsely declare a variable as significantly associated with DFS due to confounding. As demonstrated by the slopes of the models and the VIF, both overfitting and multicollinearity were not significant concerns. Finally, we opted to use bootstrapping for internal validation in preference to an alternative cohort which maximised the number of patients that could be included in the model.

There are limitations to the current study. Firstly, the present study is a single center that relies on internal validation. External validation is required to ensure that the model functions accurately in different populations and to ensure that the model does not suffer from overfitting. Furthermore, although our center had a well-defined post-operative surveillance pathway, it is possible that patients had recurrence that was not identified, out with the surveillance period. Lastly, the type of resection (e.g. complete mesocolic excision) was left to the discretion of the surgeon and accounting for such details may have improved the predictive accuracy further.

In conclusion, the present study identifies variables that are associated with long-term (≥5 year) DFS in patients who have underwent a resection for a right sided colon cancer with curative intent. The pre-and post-operative prediction tools successfully estimate the 1-, 3-, and 5-year DFS using demographic, clinical, biochemical, operative and pathological factors.

## Data Availability

Data will be made available upon reasonable request.

## Research registration unique identifying number (UIN)

Research registry (researchregistry10432).
